# ﻿*Hanseniatrifoliolata*, a new species (Apiaceae) from Shaanxi, China

**DOI:** 10.3897/phytokeys.213.83632

**Published:** 2022-11-14

**Authors:** Qiu-Ping Jiang, Megan Price, Xiang-Yi Zhang, Xing-Jin He

**Affiliations:** 1 Key Laboratory of Bio-Resources and Eco-Environment of Ministry of Education, College of Life Sciences, Sichuan University, 610065, Chengdu, Sichuan, China Sichuan University Chengdu China; 2 Sichuan Key Laboratory of Conservation Biology on Endangered Wildlife, College of Life Sciences, Sichuan University, Chengdu, 610065 Sichuan, China Sichuan University Chengdu China

**Keywords:** Apiaceae, *Hansenia*, new species, phylogenetic analyses

## Abstract

*Hanseniatrifoliolata* Q.P.Jiang & X.J.He (Apiaceae), is described as new from Shaanxi Province, northwest China. The mericarp features of *H.trifoliolata* resemble *H.himalayensis* and *H.phaea* and molecular phylogenetic analyses (combining ITS and plastid genomes data) suggest that *H.trifoliolata* is closely related to the group formed by *H.oviformis* and *H.forbesii*. The new species *H.trifoliolata* has unique 3-foliolate leaves and differ from other *Hansenia* species in its leaves, umbel numbers and size. A comprehensive description of *H.trifoliolata* is provided, including habitat environment and detailed morphological traits.

## ﻿Introduction

The Apiaceae is a large family with high morphological diversity, the generic and tribal delimitations within it being notoriously difficult (Shan and Sheh 1992; Plunkett and Downie 1999). Fruit characteristics have long been regarded as one of the most important sources of evidence for generic and tribal delimitation within the family (Drude 1898; Liu et al. 2003, 2007, 2009; Winter et al. 2008; Magee et al. 2010, 2011).

*Notopterygium* H. Boissieu (Apiaceae) was first established by Boissieu in 1903 with two species, *N.forbesii* H. Boissieu and *N.franchetii* H. Boissieu, which later appeared to be identical (Boissieu 1903). In some later studies, the genus *Notopterygium* contained six species: *N.forbesii*, *N.forrestii* H. Wolff, *N.oviforme* Shan, *N.incisum* Ting ex H. T. Chang, *N.pinnatiivolucellatum* Pu et Y. P. Wang and *N.tenuifolium* Sheh et Pu (Wolff 1930; Shan 1943; Chang 1975; Pu and Wang 1994; She and Pu 1997; Pu et al. 2000; She and Watson 2005a). The roots of *N.incisum* and *N.forbesii* are used in traditional Chinese medicine and named “Qiang Huo” (Wang et al. 1996; She and Watson 2005a; Wei et al. 2019). Although *Notopterygium* has long been treated as an endemic genus in China, Pimenov et al. (2008) merged it into the genus *Hansenia*.

*Hansenia* Turcz. belongs to the East Asia Clade of Apiaceae and it was first established by Turczaninow in 1844, with *H.mongolica* Turcz. as the type species (Turczaninow 1844; She and Watson 2005a; Pimenov et al. 2008; Downie et al. 2010; Pimenov 2017; Gou et al. 2020). *Hansenia* used to be treated as a monotypic genus and then some species had been transferred into this genus. Pimenov et al. (2008) transferred all the species of *Notopterygium*, except *N.tenuifolium*, to *Hansenia* through comparative morphological and molecular phylogenetic analyses and proposed five new combinations: *H.forbesii* (H. Boissieu) Pimenov & Kljuykov, *H.forrestii* (H. Wolff) Pimenov & Kljuykov, *H.oviformis* (R. H. Shan) Pimenov & Kljuykov, *H.weberbaueriana* (Fedde ex H. Wolff) Pimenov & Kljuykov and *H.pinnatiinvolucellata* (F. T. Pu & Y. P. Wang) Pimenov & Kljuykov (Pimenov et al. 2008; Pimenov 2017). Due to the lack of relevant material, *N.tenuifolium* was still retained in the genus *Notopterygium*. Subsequently, based on morphological and molecular data, Jia et al. (2019) considered that *H.pinnatiinvolucellata* was a synonym of *H.weberbaueriana*. Additionally, Tan et al. (2020), based on morphological characters and molecular data, transferred the two species of the genus *Haplosphaera* Handel-Mazzetti (Apiaceae) (She and Watson 2005b) into the genus *Hansenia* and proposed two new combinations: *H.himalayensis* (Ludlow) J.B. Tan & X.G. Ma and *H.phaea* (Handel-Mazzetti) J.B. Tan & X.G. Ma (Tan et al. 2020). Therefore, there are six species in *Hansenia* and one species in *Notopterygium* to date.

During a botanical expedition to Feng County in western Shaanxi Province in 2019, a umbelliferous species with thin stem and unusual 3-foliate leaves was collected. Species with 3-foliolate leaves are rare in Apioideae and only *Trachydiumtrifoliatum* H. Wolff is known in China (Shan and Sheh 1992). 3-foliolate leaves are commonly found in the genus *Sanicula* L. (Apiaceae) (She and Phillippe 2005), but the fruits were significantly different from *Sanicula*. After consulting relevant floras and literature, we identified that the fruit of the new species resembles *H.himalayensis* and *H.phaea* and further molecular phylogenetic analyses supported our conclusion. Based on careful morphological and molecular analyses, we identified it as a new species of *Hansenia*.

## ﻿Materials and methods

### ﻿DNA extraction and sequencing

Fresh leaves of *Hanseniatrifoliolata* were collected from wild plants, desiccated and stored in silica gel. The herbarium specimens were stored in the Herbarium, College of Life Sciences, Sichuan University (SZ). Specimen voucher details were provided in Table 1 and Suppl. material 1: Fig. S2. Total genomic DNA was extracted from the stored dry leaves, using a CWBIO plant genomic DNA extraction kit (CWBIO, Beijing, China), following the manufacturer’s protocols. PCR-amplification of the complete ITS region used the primers of ITS4 (5’-TCC TCCGCT TAT TGA TAT GC- 3’) and ITS5 (5’-GGA AGTAAA AGT CGT AAC AAG G-3’; White et al. 1990). PCR amplification was undertaken in a 30 μl volume reaction, containing 3 μl plant total DNA, 1.5 μl of each forward primer and reverse primer, 10 μl ddH_2_O and 15 μl 2×Taq MasterMix (CWBIO, Beijing, China). The PCR amplification of the nrITS region had an initial denaturation for 4 min at 94 °C, followed by 30 cycles of 45 s at 94 °C, 45 s at 53 °C and 60 s at 72 °C, then a final extension of 10 min at 72 °C. All PCR products were sent to Sangon (Shanghai, China) for sequencing after being examined using a 1.5% (w/v) agarose TAE gel. The DNA sequences of nrITS were applied for phylogenetic analyses and detailed information as outlined in Table 1.

**Table 1.** Voucher details and GenBank accession number of *Hanseniatrifoliolata*.

Taxa (Species number)	Voucher	Locality	Genbank number	
			Plastid genome	ITS
*Hanseniatrifoliolata*	JQP19082004	Feng County, Shannxi Province	OM281945	OM800961
				OM800962

### ﻿Plastid genome sequencing, assembly and annotation

We sequenced, assembled and annotated the plastid genome of *Hanseniatrifoliolata*, then compared it with other species of *Hansenia*. The processes of plastid genome sequencing, assembly and annotation were performed as follows.

The Illumina Novaseq 6000 platform (Illumina, San Diego, CA, USA) at Novogene (Beijing, China) was used to sequence the resultant DNA with Novaseq 150 sequencing strategy. The remaining clean data were assembled using NOVOPlasty 2.7.1 (Dierckxsens et al. 2017) with the default K-mer value 39 and rbcL of *H.oviformis* (GenBank accession No.: MF787597.1) being used as seed input. Preliminary genome annotation was conducted using PGA (Qu et al. 2019), with manual modifications for uncertain genes and uncertain start and stop codons, based on comparison with other related plastid genomes, using Geneious R11 soft (Kearse et al. 2012). Protein-coding sequence (CDS) was extracted from the plastid genome using the PhyloSuite programme (Zhang et al. 2020). The plastid genome of *H.trifoliolata* was submitted to GenBank and the accession number was listed in Table 1.

### ﻿Phylogenetic analyses

We used MEGA7 (Kumar et al. 2016) to align DNA sequences with manual adjustment to improve the accuracy of sequence alignment. Phylogenetic analyses were undertaken applying Maximum Likelihood (ML) and Bayesian Inference (BI) analyses. Based on the Akaike Information Criterion (AIC) implemented in MrModelTest version 2.2 (Nylander 2004), the best-fit nucleotide substitution models for the ITS sequences (GTR+G) and protein-coding sequences (GTR+G+I) were determined, respectively. ML analyses were undertaken using RAxML v.8.2.4 (Stamatakis 2014) with the best-fit model and 1000 bootstrap replicates. BI analyses were conducted with MrBayes version 3.2 (Ronquist et al. 2012). Four simultaneous runs were performed using Markov Chain Monte Carlo (MCMC) simulations for 10 million generations, starting from a random tree and sampling one tree every 1000 generations. The first 20% of obtained trees were discarded as burn-in and the remaining were used to calculate a majority-rule consensus topology and posterior probability (PP) values.

## ﻿Results

### ﻿Morphological study

**Figure 1.**
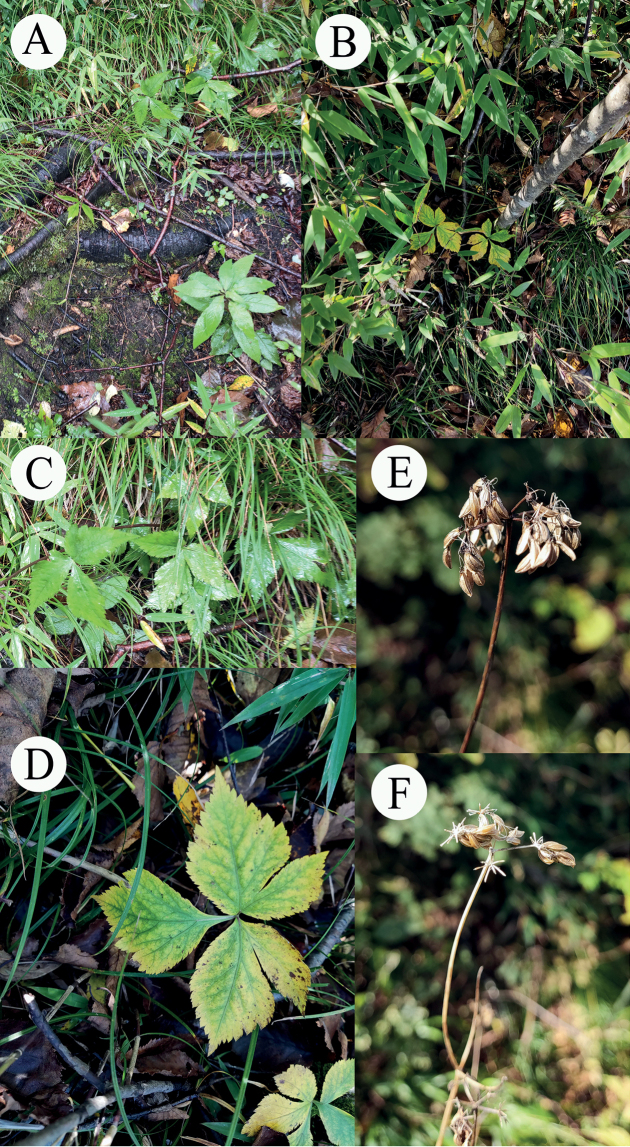
*Hanseniatrifoliolata* in the field **A, B** habitat **C** cauline leaves **D** basal leaves **E, F** umbels and fruits.

**Figure 2.**
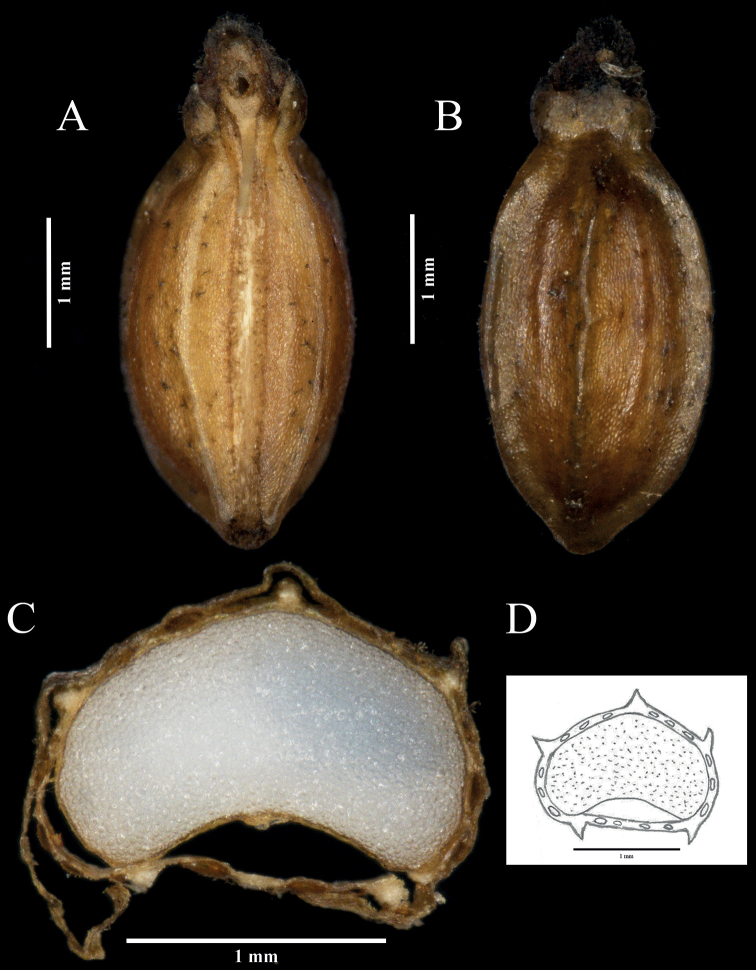
Fruit characters of *Hanseniatrifoliolata***A** commissural side of fruit **B** dorsal view of fruit **C** cross-section of fruit **D** the illustration of the fruit in transverse section. Voucher: JQP21092801.

We collected several specimens of *H.trifoliolata* from Feng County, Shannxi Province and the type locality at an elevation of 2300–2500 m (Fig. 1). After field observation, we investigated the fruit morphological characteristics of *H.trifoliolata* (Fig. 2), including fruit shape and size, ribs, vittae and endosperm which are highly similar to *H.himalayensis* and *H.phaea*. We compared the morphological characteristics of *H.trifoliolata* with the related species (*H.himalayensis*, *H.phaea*, *H.oviformis*, *H.forbesii* and *H.forrestii*), including life form, leaves, umbel rays and fruits (Table 2).

**Table 2.** Diagnostic morphological characters of *Hanseniatrifoliolata* and related species.

Character	Taxon					
	*H.trifoliolata*	*H.himalayensis*	*H.phaea*	*H.oviformis*	*H.forbesii*	*H.forrestii*
Live form	monocarpic	polycarpic	polycarpic	monocarpic	polycarpic	monocarpic
Plant height (cm)	60–90	80–120	55–90	40–60	80–180	50–100
Leaf in outline (basal)	blade broad-triangular, 3-foliolate	blade ovate-triangular, 3-pinnate	blade broad-triangular or triangular-ovate, ternate-1–2-pinnate	broadly triangular 2-pinnate	oviform 3-pinnate	broadly triangular, 2-pinnate
Median leaflets (pinnae) (basal)	cuneate-obovate or rhombic, base cuneate, with irregularly doubly serrate, apex obtuse	pinnatifid, pinnae 3–6 pairs, triangular or narrowly ovate-triangular, ultimate segments, mucronate, acute-dentate	ovate or obovate, 3-parted, base cuneate; with irregularly doubly serrate or serrate, apex obtuse	(broadly) obovate to almost round, base cuneate, margins serrulate, apex obtuse	broadly lanceolate to oviform-lanceolate, base obtuse or cuneate, margins serrate	oviform to lanceolate, base cuneate, margins irregular or sharply serrate
Lateral leaflets (pinnae) (basal)	oblique-ovate, base oblique, often shallowly or deeply uneven 2-parted or not divided; irregularly doubly serrate, apex obtuse	pinnatifid, pinnules 3–4 pairs, ultimate segments mucronate, acute-dentate	ovate to ovate-lanceolate, base oblique; with irregularly doubly serrate or serrate, apex obtuse	ovate or elliptic, base truncate; margins serrulate, apex obtuse	broadly lanceolate to oviform-lanceolate, base obtuse or cuneate; margins serrate	oviform to lanceolate, base cuneate, base oblique; margins irregular or sharply serrate
Umbels	compound umbel, 3–7-rayed, unequal	compound umbel, 2–6-rayed, subglobose, unequal	Subglobose	compound umbel, 5–9-rayed, rays very unequal	compound umbel, 11–20-rayed, rays ± equal	compound umbel, 6–9-rayed, unequal
Calyx teeth	ovate-triangular, 0.3–0.5 mm	inconspicuous, triangular, ca. 0.1 mm	ovate-triangular, 0.4 × 0.5 mm	short, triangular, ca. 0.4 mm	short, lanceolate, ca.0.5 mm	ovate-lanceolate, 0.3–0.6 mm
Fruit	obovoid-oblong or long-ellipsoid, 4–6 mm × 1.4–2.1 mm; constricted at the commissure	obovoid-oblong or long-ellipsoid, 6–7 mm × 1.5–2 mm, slightly constricted at the commissure	obovoid-oblong, 4–5 mm × 2–2.5 mm; no constricted at the commissure	globose, 4–5 × 2–3 mm; no constricted at the commissure	oblong-ellipsoid, ca. 5 × 4 mm; no constricted at the commissure	subglobose, ca. 3–3.5 × 2.5–3 mm; no constricted at the commissure
Stylopodium	conic	low-conic	depressed	flat	conic	depressed
Mericarp ribs	± equal, prominent to narrow-winged	± equal, conspicuous, narrowly winged	± equal, narrow-winged	± equal, broadly winged	± equal, winged	± equal, winged
Endosperm (at commissural side)	concave	deeply concave	concave	slightly concave	broadly and not deeply concave	concave
Vittae in dorsal furrows	3 (4)	3	3	1–2	2–4	3
Vittae in commissure	2–5	6	4–6	4	4–5	4–6

### ﻿Phylogenetic analyses

**Figure 3.**
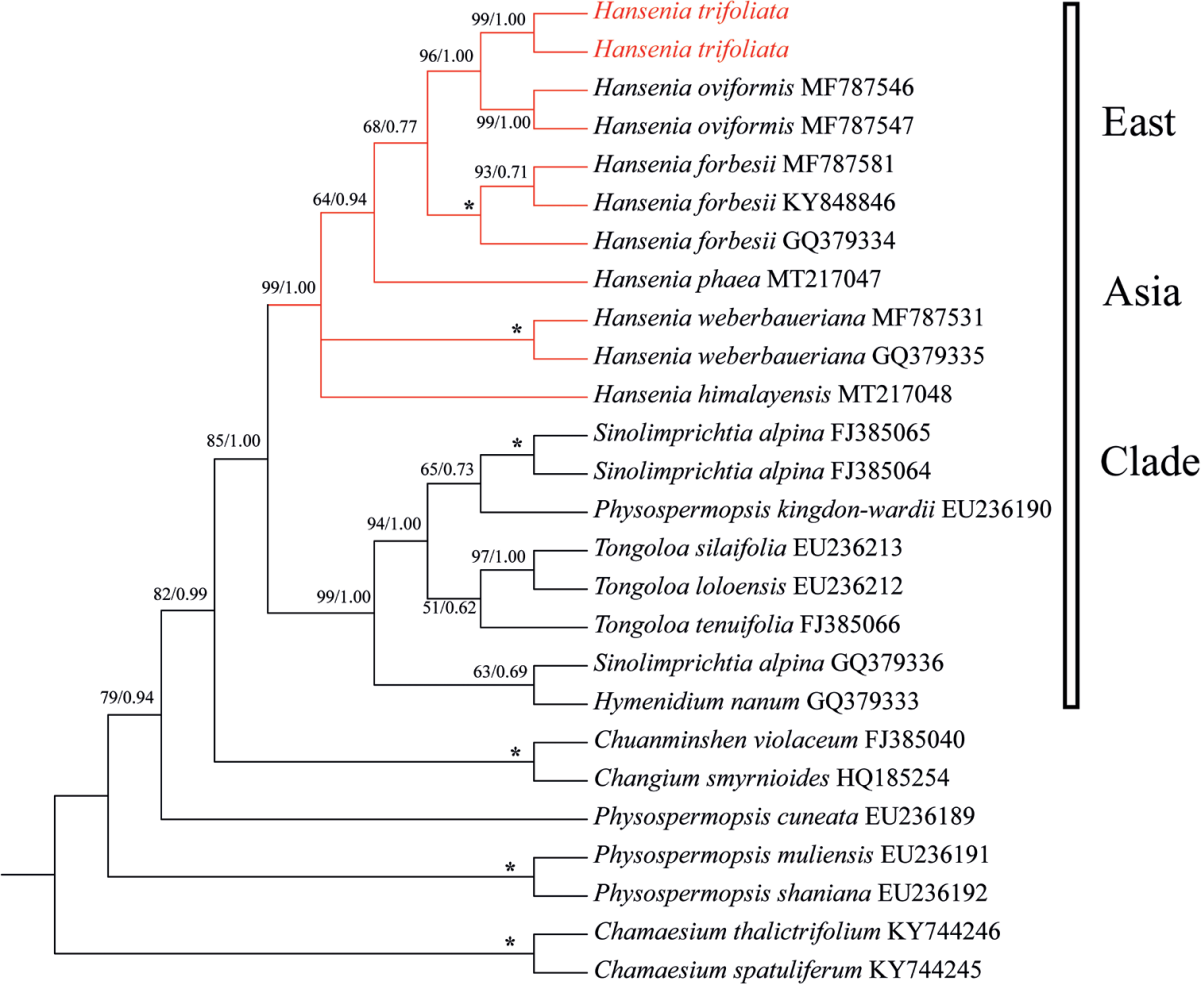
Bayesian 50% majority-rule consensus tree of *Hanseniatrifoliolata*, other species of *Hansenia* and related species inferred from ITS sequences using a GTR+G nucleotide substitution model. The tree is rooted with two species of *Chamaesium*. Maximum Likelihood bootstrap support (ML BS) and Bayesian posterior probabilities (BIPP) are presented at the nodes, * representing the best support (100%). The ITS sequences obtained from NCBI exhibited the GenBank number adjacent to the species names.

The phylogenetic analysis result, based on ITS data, is shown in Fig. 3. The details of the ITS dataset that we sequenced for phylogenetic analysis are listed in Table 1. The phylogenetic trees derived from BI and ML analyses were topologically consistent. Thus, only the BI tree is shown in Fig. 3, with bootstrap support values obtained from ML analyses. The phylogenetic tree showed that *H.trifoliolata* was sister to *H.oviformis*, with strong support (Bayesian inference posterior probability, BI = 1.00; maximum parsimony bootstrap, ML = 96%). Additionally, *H.trifoliolata* and other *Hansenia* species formed a monophyletic group with the support very close to maximum (BI = 1.00; ML = 99%).

**Figure 4.**
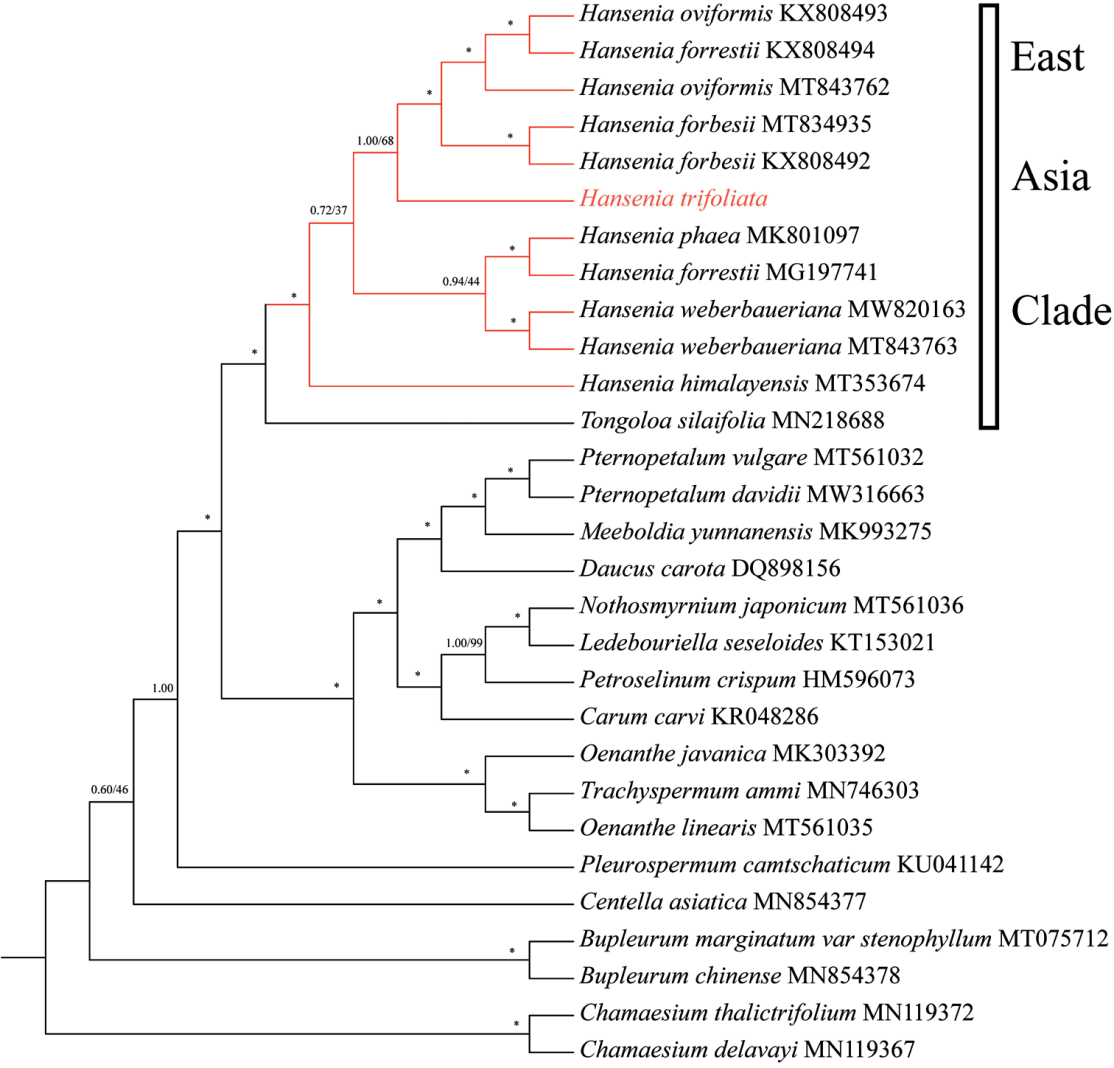
Bayesian 50% majority-rule consensus tree of *Hanseniatrifoliolata*, other species of *Hansenia* and related species inferred from protein-coding genes of plastid genomes using a GTR+G+I nucleotide substitution model. The tree is rooted with two species of *Chamaesium*. Maximum Likelihood bootstrap support (ML BS) and Bayesian posterior probabilities (BIPP) are presented at the nodes,* representing the best support (100%). The plastid genome sequences obtained from NCBI exhibited the GenBank number adjacent to the species names.

The result of the phylogenetic analysis, based on the plastid genome data, is shown in Fig. 4. The plastid genome GenBank number of *H.trifoliolata* is listed in Table 1. The phylogenetic trees derived from BI and ML analyses were topologically consistent. Therefore, only the BI tree is shown in Fig. 4, with bootstrap support values obtained from ML analyses. The phylogenetic tree showed that *H.trifoliolata* clustered with the communities of *H.oviformis* and *H.forbesii* (BI = 1.00; ML = 68%). This is the same as the ITS tree, with *H.trifoliolata* and other *Hansenia* species forming a monophyletic group with maximum support (BI = 1.00; ML = 100%).

## ﻿Discussion

The fruits of *H.trifoliolata* were similar to *H.himalayensis* and *H.phaea* in fruit shape and size, mericarp ribs and both vittae in dorsal furrows and in the commissure. Additionally, the endosperm (at the commissural side), slightly or deeply concave, was common in *Hansenia* (Pimenov et al. 2008; Tan et al. 2020). The fruit shape of *Hansenia* can be divided into two groups by the shape and ribs: fruits oblong-ellipsoid, subglobose or globose, all ribs winged or broadly winged ribs (including *H.forrestii*, *H.forbesii*, *H.mongolica*, *H.oviformis* and *H.weberbaueriana*); fruits obovoid-oblong or long-ellipsoid, ribs prominent to narrowly winged (including *H.trifoliolata*, *H.himalayensis* and *H.phaea*). All species’ fruit ribs are 5 and ± equal, except for *H.weberbaueriana* where the ribs are 3–5, equal or a little unequal (Jia et al. 2019). Moreover, there is a constriction at the commissure in *H.trifoliolata*, with a similar phenomenon being found in *H.himalayensis* that has a slight constriction at the commissure (She and Watson 2005a; Tan et al. 2020).

The life form of *H.trifoliolata* is monocarpic, which is uncommon in *Hansenia*, except for *H.forrestii* which seems to be similar (Pimenov et al. 2008). Through observation of the specimens of *H.oviformis*, we believed that *H.oviformis* is monocarpic. The leaves of *Hansenia* species are often 1–3-pinnate, leaflets pinnatifid (including *H.mongolica*, *H.himalayensis* and *H.weberbaueriana*) or leaflets not pinnatifid (including *H.forrestii*, *H.forbesii*, *H.oviformis*, *H.phaea* and *H.trifoliolata*). The stylopodium shape in the genus is continuous, from depressed to flat, to low-conic and conic. All species of *Hansenia* display compound umbels, except for *H.phaea* and the number of rays are either below ten (including *H.trifoliolata*, *H.himalayensis*, *H.oviformis* and *H.forrestii*) or ten to twenty (including *H.forbesii*, *H.mongolica* and *H.weberbaueriana*) (Pimenov et al. 2008; Tan et al. 2020).

In our phylogenetic analyses, *H.trifoliolata* and other *Hansenia* species formed a monophyletic group in both ITS and plastid trees with very strong support (ITS trees: BI = 1.00, ML = 99%; plastid trees: BI = 1.00, ML = 100%). Though the position of *H.trifoliolata* within *Hansenia* had a slight difference between ITS trees and plastid trees (ITS trees: *H.trifoliolata* was sister to *H.oviformis*, then clusters with *H.forbesii*; plastid trees: *H.trifoliolata* clustered with the communities of *H.oviformis* and *H.forbesii*), there is no doubt that *H.trifoliolata* is a member of the genus *Hansenia*.

*H.trifoliolata* overlaps in its distribution with *H.forbesii* and *H.weberbaueriana* in the western Shaanxi Province and south-eastern Gansu Province.

The molecular data and morphological evidence strongly support the circumscription of *H.trifoliolata* as a new species belonging to *Hansenia*.

### ﻿Key to the species of *Hansenia*

**Table:** 

1a	Fruit oblong-ellipsoid, subglobose or globose or elliptic, all ribs winged or broadly winged, wings equal or unequal	**2**
2a	Rays below ten, unequal	**3**
3a	Ultimate leaf segments ovate-lanceolate, 2.5–8 cm; bracteoles linear, shorter than flowers	***H.forrestii***
3b	Ultimate leaf segments ovate, 1.5–3.5 cm; bracteoles filiform, longer than flowers	***H.oviformis***
2b	Rays ten to twenty, ± equal	**4**
4a	Leaves pinnatisect, leaflets pinnatifid	**5**
5a	Bracteoles linear or pinnatifid, fruit ribs 3–5, ultimate leaf segments oblong, margin pinnatifid or variously laciniate-dentate	***H.weberbaueriana***
5b	Bracteoles linear, ribs 5, ultimate leaf segments broadly ovate to oblong, at the margin toothed, teeth obtuse	***H.mongolica***
4b	Leaves pinnate, leaflets not pinnatifid, ultimate leaf segments ovate to oblong-ovate, margin entire or coarsely toothed	***H.forbesii***
1b	Fruit obovoid-oblong or long-ellipsoid, ribs prominent to narrowly winged	**6**
6a	Basal leaves and cauline leaves 3-foliolate, umbels 2–5 cm across, rays unequal	***H.trifoliolata***
6b	Basal leaves ternate-1–3-pinnate, flowers densely crowded into a compact, globose heads	**7**
7a	Basal leaves ternate-1–2-pinnate; petals obovate, apex narrowly inflexed	***H.phaea***
7b	Basal leaves 3-pinnate; petals broad-ovate, spoon-shaped apex acute	***H.himalayensis***

### ﻿Description of the new species

#### ﻿ Hansenia trifoliolata

Taxon classificationPlantaeApialesApiaceae

Q.P.Jiang & X.J.He sp. nov.

66BD5D75-34F8-54C7-88FC-DC2BF3CFF1F5

urn:lsid:ipni.org:names:77307988-1

**Figure 5.**
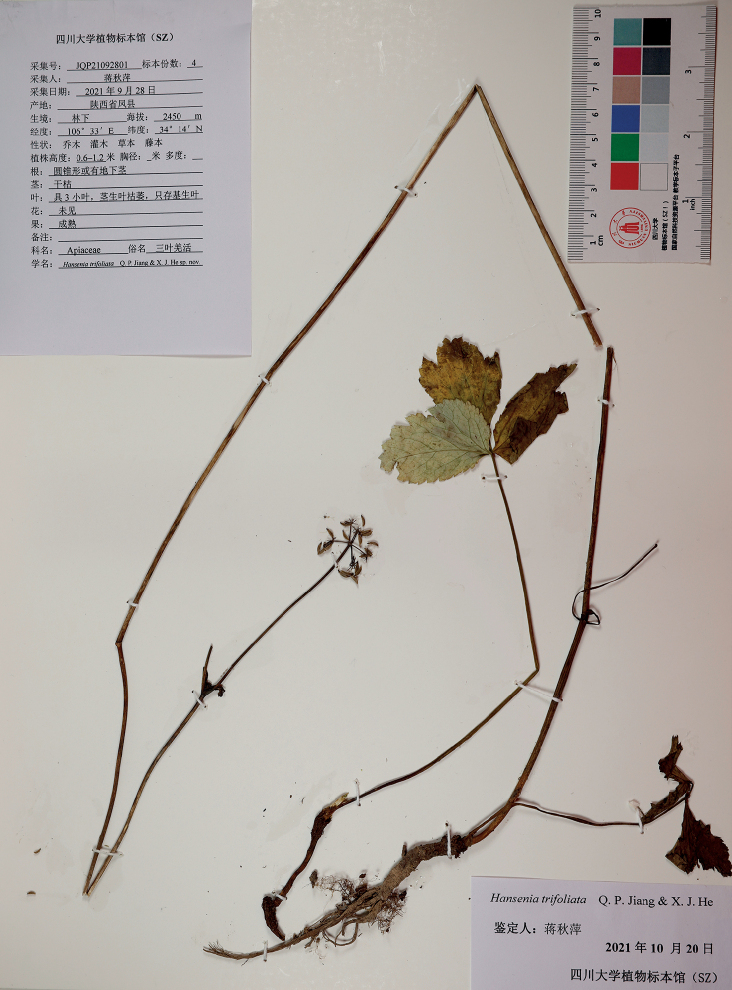
Holotype of *Hanseniatrifoliolata*, fruiting. Vouchers: JQP21092801.

Figs 1 , 5 ; Suppl. material 1: Fig. S2

##### Diagnostic characters.

Monocarpic. Root cylindrical, branched or partial rhizomes. Leaves 3-foliolate. Umbels 2–5 cm across, rays 3–7, unequal. Stylopodium conical. Fruits are obovoid-oblong or long-ellipsoid, have 5 ribs, ribs prominent to narrow-winged and endosperm (at commissural side) concave. It is clearly distinguished from *H.phaea* and *H.himalayensis* in leaves (ternate-1–2-pinnate and 3-pinnate vs. 3-foliolate). Compared to other *Hansenia* species (i.e. *H.forrestii*, *H.oviformis* and *H.forbesii*), *H.trifoliolata* also shows distinctive morphological characters, especially in fruits characters (shape and ribs) and leaves (3-foliolate is unique in *Hansenia*).

##### Type.

China, Shaanxi Province: Tongtianhe National Forest Park, Feng County, elevation 2430 m a.s.l., 34°14'N, 106°33'E, 28 Sep 2021, Q. P. Jiang, JQP21092801, fruiting (Holotype: SZ).

##### Description.

Biennial, herb, 60–90 cm high. Root cylindrical, branched or partial rhizomes. Stem purplish-green, thinly ribbed, glabrous, thin. Leaves 3-foliolate, green, blade broad-triangular, irregularly doubly serrate, teeth mucronate; central leaflets cuneate-obovate or rhombic, 4–6 × 2–3.5 cm, with irregularly doubly serrate, base cuneate; lateral leaflets oblique-ovate, base oblique, often shallowly or deeply uneven 2-parted or not divided, 2–5 × 3.5–6.5 cm. Basal petioles 15–20 cm, petioles shorten upwards; sheaths narrow-oblong, glabrous, with margin irregularly coarse-cuspidate-serrate. Umbels 20–50 mm across; peduncles 5–20 mm long, glabrous; bracts 0 to 2, linear; rays 3 to 7, 5–25 mm long, glabrous; bracteoles 2 to 7, linear, 3–8 mm long; raylets 5 to 11, 1–3 mm long. Flowers unknown; calyx teeth ovate-triangular, 0.3–0.5 mm; petals unknown; stylopodium conical. Fruit obovoid-oblong or long-ellipsoid, 1.4–2.1 × 4–6 mm; mericarps 5-ribbed, ribs prominent to narrow-winged; vittae 3 (4) in each furrow, 2–5 on commissure; endosperm (at commissural side) concave, commissure width 0.8–1.35 mm.

##### Etymology.

The specific epithet refers to the distinctive 3-foliolate leaves.

##### Phenology.

Flowering from July to August, and fruiting from August to September.

##### Distribution, habitat and ecology.

At present, this new species has only been found in the type locality in Tongtianhe National Forest Park, Feng County, Shaanxi Province, China. According to the growing environment, we speculate it may inhabit forests at an elevation of 2300 m to 2500 m in western Shaanxi Province and south-eastern Gansu Province. This new species grows in humid environments under the forests.

##### Additional specimens examined

**(paratypes).** China: Shaanxi Province, Baoji City, Feng County, Tongtianhe National Forest Park, elevation 2430 m a.s.l., 34°14'N, 106°33'E, 20 Aug 2019, Q. P. Jiang and X. Y. Zhang, JQP19082004 (photo SZ !).

## Supplementary Material

XML Treatment for Hansenia trifoliolata

## Supplementary materials

Supplementary material 1 Figure S1, S2 Data type Docx file. Explanation note **Figure S1.** Fruit of *Hanseniatrifoliolata*. **Figure S2.** Isotype of *Hanseniatrifoliolata* and paratype of *H.trifoliolata*. https://binary.pensoft.net/file/769334This dataset is made available under the Open Database License (http://opendatacommons.org/licenses/odbl/1.0/). The Open Database License (ODbL) is a license agreement intended to allow users to freely share, modify, and use this Dataset while maintaining this same freedom for others, provided that the original source and author(s) are credited.Qiu-Ping Jiang, Megan Price, Xiang-Yi Zhang, Xing-Jin He
