# Supplementary material for: ﻿Hanseniatrifoliolata, a new species (Apiaceae) from Shaanxi, China
Source: PhytoKeys. 2022 Nov 14;213:79–93. doi: 10.3897/phytokeys.213.83632 (PMC9836542; doi:10.3897/phytokeys.213.83632)
Supplement: Supplementary material 1 — Figure S1, S2 [file phytokeys-213-079_article-83632__-s001.docx]

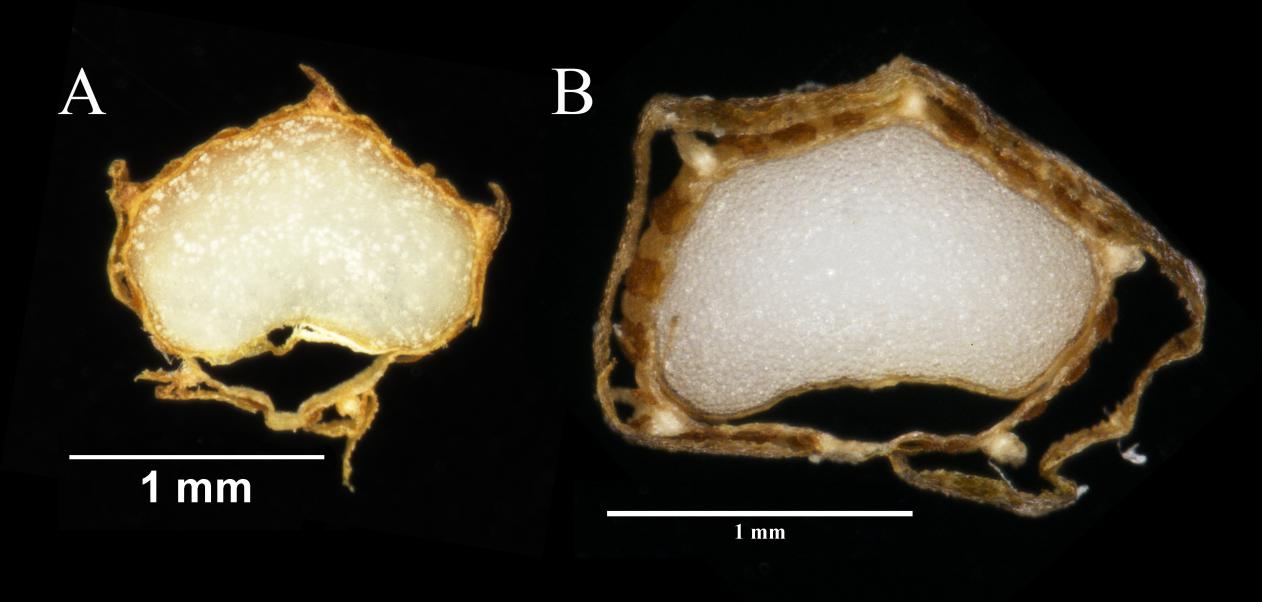


**Figure S1**. Fruit of *Hansenia trifoliata*: (A) narrow-winged; (B) with 5 vittae in commissural.


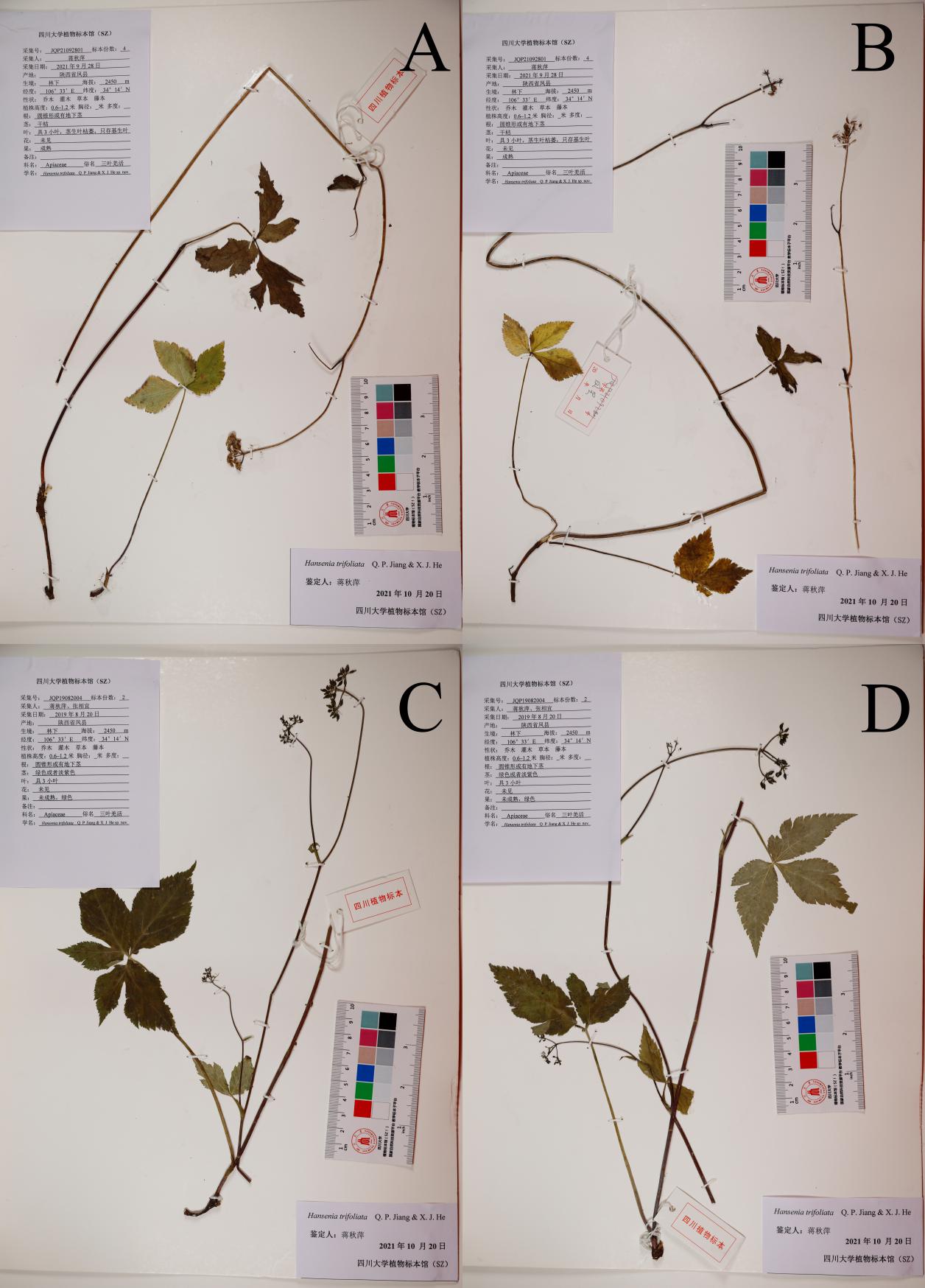


**Figure S2.** (A) and (B): isotype of *Hansenia trifoliata*, vouchers: JQP21092801. (C) and (D): paratype of *H. trifoliata*, vouchers: JQP19082004.
